# Plasma cytokines in women with chronic fatigue syndrome

**DOI:** 10.1186/1479-5876-7-96

**Published:** 2009-11-12

**Authors:** Mary Ann Fletcher, Xiao Rong Zeng, Zachary Barnes, Silvina Levis, Nancy G Klimas

**Affiliations:** 1 Department of Medicine, University of Miami Miller School of Medicine, 1600 NW 10th Ave, Miami, FL USA; 2 Miami Veterans Health Care Center, 1201 NW 16th St, Miami, FL USA

## Abstract

**Background:**

Chronic Fatigue Syndrome (CFS) studies from our laboratory and others have described cytokine abnormalities. Other studies reported no difference between CFS and controls. However, methodologies varied widely and few studies measured more than 4 or 5 cytokines. Multiplex technology permits the determination of cytokines for a large panel of cytokines simultaneously with high sensitivity and with only 30 ul of plasma per sample. No widely accepted laboratory test or marker is available for the diagnosis or prognosis of CFS. This study screened plasma factors to identify circulating biomarkers associated with CFS.

**Methods:**

Cytokines were measured in plasma from female CFS cases and female healthy controls. Multiplex technology provided profiles of 16 plasma factors including the pro -inflammatory cytokines: tumor necrosis factor α (TNFα), lymphotoxin α (LTα), interleukin (IL) - IL-Iα, IL-1β, IL-6; T_H_1 cytokines: interferon γ (IFNγ), IL-12p70, IL-2, IL-15; T_H_2: IL-4, IL-5; T_H_17 cytokines, IL-17 and IL-23; anti-inflammatory cytokines IL-10, IL-13; the inflammatory mediator and neutrophil attracting chemokine IL-8 (CXCL8). Analysis by receiver operating characteristic (ROC) curve assessed the biomarker potential of each cytokine.

**Results:**

The following cytokines were elevated in CFS compared to controls: LTα, IL-1α, IL-1β, IL-4, IL-5, IL-6 and IL-12. The following cytokines were decreased in CFS: IL-8, IL-13 and IL-15. The following cytokines were not different: TNFα, IFNγ, IL-2, IL-10, IL-23 and IL-17. Applying (ROC) curve analyses, areas under the curves (AUC) for IL-5 (0. 84), LTα (0.77), IL-4 (0.77), IL-12 (0.76) indicated good biomarker potential. The AUC of IL-6 (0.73), IL-15 (0.73), IL-8 (0.69), IL-13 (0.68) IL-1α (0.62), IL-1β (0.62) showed fair potential as biomarkers.

**Conclusion:**

Cytokine abnormalities are common in CFS. In this study, 10 of 16 cytokines examined showed good to fair promise as biomarkers. However, the cytokine changes observed are likely to more indicative of immune activation and inflammation, rather than specific for CFS. As such, they are targets for herapeutic strategies. Newer techniques allow evaluation of large panels of cytokines in a cost effective fashion.

## Background

According to a Centers for Disease Control (CDC) report [1] the overall prevalence in the USA of Chronic Fatigue Syndrome (CFS), is 235 per 100,000 persons (95% confidence interval, 142-327 per 100,000 persons). Up to 80% of those affected are women [2]. These individuals suffer from severe fatigue that impairs daily activity, diminishes quality of life for years and has no known cure [3]. CFS represents an economic burden for society (e.g., high rates of unemployment due to disability) and healthcare institutions [4]. Hypothetical initiating events for CFS include infections, psychiatric trauma and exposure to toxins. Many of the symptoms are inflammatory in nature (myalgia, arthralgia, sore throat, tender lymphadenopathy), and have prompted a theory of infection induced illness [5,6]. In 60 to 80% of published samples, CFS presents with acute onset of illness, with systemic symptoms similar to influenza infection that do not subside [7]. These observations have led to reports of associated microbial infections or reactivation of latent viral infections [5,8-13]. However, there is no consensus as to etiology.

There is a considerable literature describing immune dysfunction in CFS [14,15]. Elevation of pro-inflammatory cytokines [16,17] and evidence of T_H_2 (T helper cell type 2) cytokine activation [15,18] were reported. Other studies reported no difference between CFS and controls. However, methodologies varied widely and few studies measured more than four or five cytokines. Lack of sensitivity of standard ELISA (enzyme-linked immunosorbent assay) technology limited use of plasma for the detection of case/control differences.

Despite evidences of immunological and molecular mediators, no individual marker or combination of markers has been sufficiently associated with CFS to enable its use as a biomarker for the diagnosis or management of CFS. The goal of this study was to determine if, using new technology, plasma cytokines had sufficient sensitivity and specificity to distinguish CFS cases from age-matched healthy controls. Using a multiplex assay, 16 cytokines (T_H_1, T_H_2, T_H_17, pro-inflammatory, anti-inflammatory) were compared among cases and controls. Because of the strong gender bias in CFS (80% female), only women were included in the study.

## Methods

### Patients

Female CFS patients (n = 40; mean age 50) were from the CFS and Related Disorders Clinic at the University of Miami. A diagnosis of CFS was made using the International Case Definition [19,20]. Female healthy controls (n = 59; mean age 53) were from a NIH funded study. All subjects signed an informed consent approved by the Institutional Review Board of the University of Miami. All CFS study subjects had a SF-36 summary physical score (PCS) below the 50^th ^percentile, based on population norms. Exclusion criteria for CFS included all of those listed in the current Centers for Disease Control (CDC) CFS case definition, including the listed psychiatric exclusions, as clarified in the International CFS Working Group [20]. All CFS subjects were assessed for psychiatric diagnosis at the time of recruitment with the Composite International Diagnostic Instrument [21]. Based on this assessment, we excluded subjects with DSM IV diagnoses for psychotic or melancholic depression, panic attacks, substance dependency, or psychoses as well as any subjects currently suicidal. We also excluded subjects with Borderline or Antisocial Personality Disorder. Subjects had no history of heart disease, COPD, malignancy, or other systemic disorders that would be exclusionary, as clarified by Reeves et al. [20]. Subjects were also excluded for the following reasons: less than 18 yrs of age, active smoking or alcohol history, history of significant inability to keep scheduled clinic appointments in past.

### Ethical Issues

This study was approved by the institutional review board and all patients gave written, informed consent.

### Blood Collection

Morning blood samples were collected into ethylene diamine tetra acetic acid. Plasma was separated within 2 hours of collection and stored at -80°C until assayed.

### Cytokine Array System

We measured 16 cytokines in plasma using Quansys reagents and instrument (Quansys Biosciences, Logan, Utah). The Quansys Imager, driven by an 8.4 megapixel Canon 20D digital SLR camera, supports 96 well plate based chemiluminescent imaging. The Q-Plex™ Human Cytokine - Screen (16-plex) is a quantitative ELISA-based test where sixteen distinct capture antibodies have been absorbed to each well of a 96-well plate in a defined array. Manipulation of the range of the standard curves and exposure time allowed reliable co mparisons between CFS patients and controls of both low and high level cytokine concentrations in plasma. For the standard curves, we used the second order (k = 2) polynomial regression model (parabolic curve), Y = b_0_+b_1_X+b_2_X^2^....+b_k_X^k^, where Y caret is the predicted outcome value for the polynomial model with regression coefficients b_1 _to k for each degree and y intercept b_0_. Quadruplicate determinations were made, i.e., each sample was run in duplicate in two separate assays.

### Statistical Analysis

The cytokine measurements were not normally distributed. Since the sample sizes between control and test groups were also different, the nonparametric Kruskal-Wallis one-way analysis based on rank sums was used to determine the magnitudes of between-group differences. Values of p < 0.05 were considered statistically significant. The diagnostic accuracy of those cytokines significantly different among cases and controls was analyzed by receiver operating characteristics (ROC) curve analyses [22] using the Statistical Package for Social Sciences (SPSS) version 16 for Windows.

## Results

We clustered the results of the cytokine assays into 5 groups according to the cytokine literature. The results of the individual Kruskal-Wallis analyses are shown in Table 1.

**Table 1:** Cytokines in Plasma of Female CFS Patients Compared to Female Healthy Controls

CYTOKINE^B^	TYPE	CFS CASES N = 40	CONTROLS N = 59	% DIFFERENCE IN MEDIAN VALUES^C^	KRUSKAL-WALLIS	
					**χ^2^**	**P**

TNFα	Pro-inflammatory	7.3 (3.4 - 22.6)	6.4 (4.5 - 38.3)	+ 14	0.0	.949

LTα	Pro-inflammatory	7.5 (4.5 - 38.3)	2.1 (4.5 - 12.4)	+ 257	20.4	.000

IL-6	Pro-inflammatory	6.4 (3.8 - 14.4)	3.2 (2.1 - 5.9)	+100	15.1	.000

IL-1α	Pro-inflammatory	3.2 (1.7 - 4.4)	2.3 (0.9 - 3.9)	+ 39	4.1	.044

IL-1β	Pro-inflammatory	13.4 (4.5 - 38.3)	6.2 (4.2 - 38.3)	+ 100	4.2	.041

IFNγ	T_H_1	3.1 (0.1 - 11.8)	2.6 (1.2 - 10.6)	+ 19	0.5	.467

IL-2	T_H_1	2.3 (1.4 - 5.4)	2.5 (2.1 - 3.5)	- 8	0.6	.420

IL-12	T_H_1	4.4 (2.4 - 7.3)	2.0 (1.7 - 2.5)	+ 120	18.8	.000

IL-15	T_H_1	13.5 (7.0 - 23.6)	27.4 (19.7 - 49.4)	- 51	15.0	.000

IL-17	T_H_17	3.8 (0.8 - 7.2)	2.9 (1.9 - 6.7)	+ 31	0.1	.785

IL-23	T_H_17	82.(70.3 - 113)	101.7 (45.0 - 375.6)	- 16	0.8	.814

IL-4	T_H_2	1.7 (0.9 - 4.3)	0.5 (.03 - 1.1)	+ 240	20.7	.000

IL-5	T_H_2	7.4 (6.3 - 10.0)	3.8 (3.2 - 5.6)	+ 95	33.6	.000

IL-10	Anti-inflammatory	3.3 (2.1 - 5.6)	3.6 (2.2 - 6.4)	- 9	0.1	.748

IL-13	Anti-inflammatory	1.7 (1.2 - 2.1)	2.0 (1.9 - 2.1)	-15	9.6	.002

IL-8 (CXCL8)	NK cell attracting	9. (5.0 - 15.8)	15.4 (11.5 - 22.2)	- 42	9.7	.002

^a ^Values are expressed as medians. Values in parentheses are 25^th ^and 75^th ^percentiles.

^b ^Cytokines determined as pg/ml.

^c ^Percent differences were calculated by using the normal controls as a reference; the + or - sign indicates the direction of change.

### Pro- inflammatory cytokines

A significant elevation in the relative amounts of 4 of 5 pro-inflammatory cytokines in peripheral blood plasma of patients with CFS was found when compared with the controls Only tumor necrosis factor (TNF)α was unchanged. In cases, lymphotoxin (LT)α was elevated by 257% and IL-6 by 100% over the controls.

### T_H_2 cytokines

Both interleukin (IL)-4 and IL-5 were elevated in CFS, with the median of IL-4 240% and of IL-5 95% higher in cases over controls.

### Anti-inflammatory cytokines

IL-13 was significantly lower (!5%) in CFS patients while IL-10 was not different.

### T_H_1 cytokines

Median plasma levels of IL-2 and IFNγ in CFS were similar to those in controls. However, IL-12 was significantly elevated (120%) and IL-15 decreased 15% in cases compared to controls.

### IL-8 (CXCL8)

This chemokine was 42% lower in the CFS patients.

### T_H_17 cytokines

IL-17 and IL-23 were not significantly different in CFS cases compared to controls.

### ROC curve analyses

**Figure 1:**
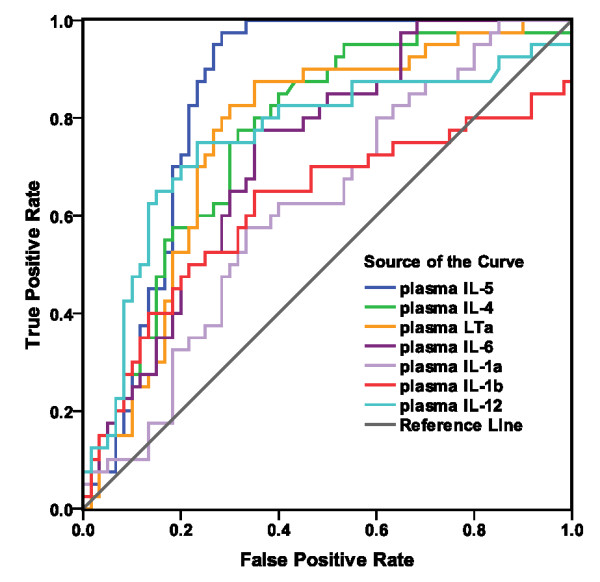
**ROC curves shows the classification performance of plasma cytokines from CFS cases and healthy controls**. Curves are for the 7 cytokines significantly elevated (p < .05) in cases compared to controls (IL-4, IL-5, IL-12, LTα, IL-1α, IL-1β, and IL-6).

**Figure 2:**
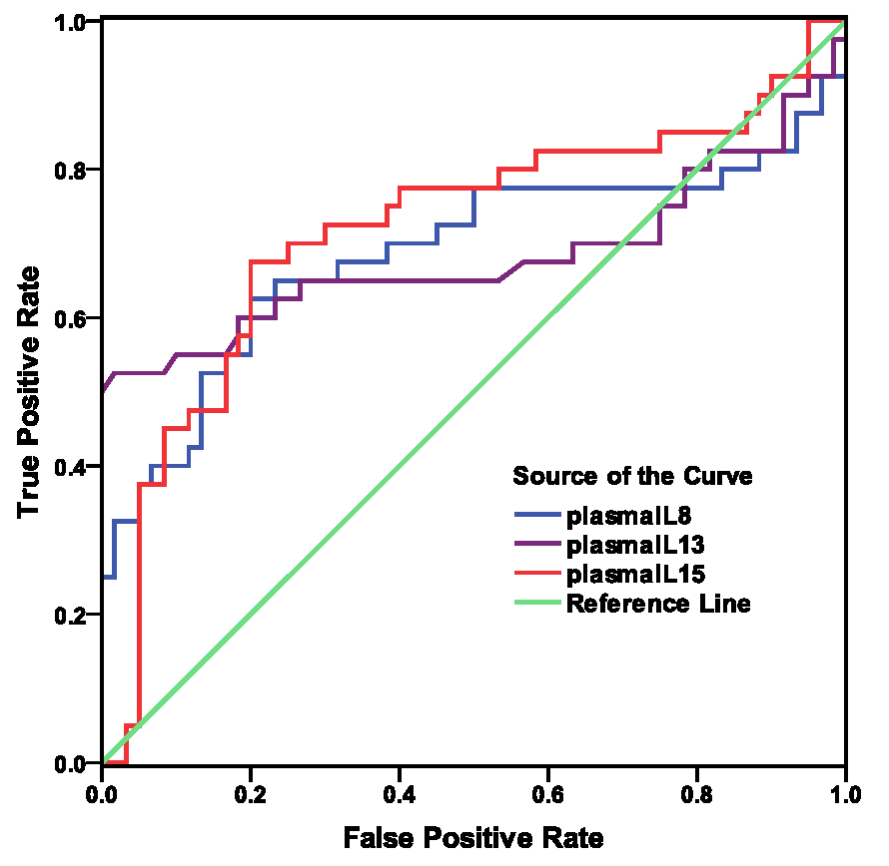
**ROC curves show the classification performance of plasma cytokines from CFS cases and healthy controls**. Curves are for the 3 cytokines significantly lower (p < .05) in cases compared to controls (IL-8, IL-13 and IL-15).

Results for those cytokines that were significantly higher in the case/control comparison are shown in Figure 1 and Table 2. Those for cytokines that were lower in CFS than controls are shown in Figure 2 and Table 3. Area under the curve (AUC) for IL-5 (0. 84), LTα (0.77), IL-4 (0.77), IL-12 (0.76) indicated good biomarker potential. Coordinates of the curves for these 4 cytokines are in Additional File 1. The AUC of IL-6 (0.73), IL-15 (0.73), IL-8 (0.69), IL-13 (0.68) IL-1α (0.62), IL-1β (0.62) showed fair potential as biomarkers (Tables 2 and 3).

**Table 2:** AUC for Plasma Cytokines Significantly Higher in CFS Cases vs. Controls

Cytokines	Area	Std. Error^a^	Asymptotic Sig.^b^	Asymptotic 95% Confidence Interval	
				Lower Boundary	Upper Boundary
**LTα**	.769	.049	.000	.673	.865
**IL-6**	.731	.050	.000	.633	.828
**IL-1α**	.620	.056	.044	.509	.730
**IL-1 β**	.621	.062	.041	.499	.744
**IL-5**	.844	.041	.000	.764	.925
**IL-4**	.770	.048	.000	.676	.864
**IL-12**	.758	.054	.000	.653	.863

^a ^Under the nonparametric assumption

^b ^Null hypothesis: true area = 0.5

**Table 3:** AUC for Plasma Cytokines Significantly Lower in CFS Cases vs. Controls

Cytokines	Area	Std. Error^a^	Asymptotic Sig.^b^	Asymptotic 95% Confidence Interval	
				Lower Boundary	Upper Boundary
**IL-8**	.685	.062	.002	.564	.806
**IL-15**	.731	.056	.000	.620	.841
**IL-13**	.682	.064	.002	.556	.808

^a ^Under the nonparametric assumption

^b ^Null hypothesis: true area = 0.5

## Discussion

Several studies report cytokine abnormalities in CFS; however, the findings are mixed. Differences between reports may be largely due to differences in methodologies [14]. Amounts of cytokines in plasma or serum are often below the level of detection in traditional ELISA assays. In addition to assay sensitivity, results using the direct approach are influenced by length of time following blood draw to separation of serum or plasma, temperature of storage and repeated thawing and freezing. *In vitro *stimulation whole blood or peripheral blood mononuclear cells (PBMC) is another approach to study cytokines. ELISA is then used to measure cytokine content of supernatants of culture fluids. Obviously, results depend on culture conditions and stimulants used. Other techniques include either in unstimulated or stimulated PBMC. Results obtained with these methodologies are not directly comparable.

The availability of sensitive multiplex technology permitted the determination of 16 cytokines simultaneously on plasma samples from female CFS patients and age and gender matched healthy controls. In the CFS cases, we found an unusual pattern of the cytokines that define the CD4 T cell. Dendritic cell derived IL-12, the main T_H_1-inducing cytokine leading to production of IFNγ, IL-2 and TNFα, was elevated. However, IFNγ, IL-2 and TNFα were unchanged in plasma of CFS cases compared to controls. Another dendritic cell derived cytokine, IL-15, was decreased. IL-2 and IL-15 are key participants in CD8 T cell and NK cell activation and function. Sharing the beta and gamma receptor subunits results in several common functions: e.g. cytotoxicity. On the other hand, due to their distinct alpha receptor subunits, they play opposing roles in immune processes such as activation induced cell death (IL-2) and immunological memory (IL-15) [23]. IL-23 (unchanged between controls and cases) stimulates the differentiation and function of the T_H_17 subset of CD4 T cells, a relatively newly described immune defense. The T_H_17 CD4 cell produces IL-17, protects surfaces (e.g., skin, lining of the intestine) against bacteria, and plays a critical role in chronic intestinal inflammation [24,25]. The unchanged IL-17 and IL-23 levels in CFS noted in this study would argue against bacterial gastrointestinal infections as playing an important role in persistent illness.

Along with the T_H_1 abnormalities, we found up regulation of T_H_2 associated cytokines, IL-4 and IL-5, in the CFS subjects. Allergy is common in CFS cases. Years ago, Straus et al, reported >50% atopy in 24 CFS patients [26]. The elevation of these two cytokines implies a type 2 shift - and diminished stimulus for cytotoxic lymphocyte function.

The probability of chronic inflammation [17] in CFS is supported by the elevation of four members of the pro-inflammatory cytokine cascade [27], LTα, IL-1α, IL-1β, and IL-6, in the CFS samples compared to controls. The exception was TNFα, although the median value for cases was 14% higher than controls and about 1/4 of CFS patients in other studies had elevated TNFα [15,17]. Interleukin-13, associated with inhibitory effects on inflammatory cytokine production, was lower in cases compared to controls. The anti-inflammatory cytokine, IL10, was not different. The inflammatory mediator IL-8 (a chemokine known as CXCL8) known to be responsible for the migration and activation of neutrophils and NK cells [28] was decreased in plasma of CFS patients.

The observations of abnormal cytokine patterns in CFS patients support the reports of retrovirus infections and reactivation of latent herpes virus infections. DeFreitas, et al found HTLV-II- like gag sequences by polymerase chain reaction and in situ hybridization as well as antibodies reactive with human T- lymphotropic virus (HTLV) in a majority of 30 CFS cases. Twenty healthy controls were negative for the three assays [11]. Holmes, et al, reported that structures consistent with stages of a Lentivirus replicative cycle were observed by electron microscopy in 12-day PBMC cultures from 10 of 17 CFS patients and not in controls [12]. Recently, DNA from a human gammaretrovirus, xenotropic murine leukemia virus-related virus (XMRV), was found in the PBMC of 68 of 101 patients compared to 8 of 218 healthy controls. Patient-derived, activated PBMC produced infectious XMRV *in vitro*. Both cell associated and cell-free transmission of the virus to uninfected primary lymphocytes and indicator cell lines was possible [13]. The XMRV *gag *and *env *sequences discovered in CFS cases were more than 99% similar to those previously reported for prostate tumor-associated strains of XMRV [29].

Latent herpes virus infections are likely to be important in CFS. Immunologic effects of persistent herpetic infections do not require of virus DNA synthesis. For example, Glazer and colleagues [9] reported that EBV encoded deoxyuridine triphosphate nucleotidohydrolase (dUTPase) upregulated the production of proinflammatory cytokines, including IL-1β and IL-6. Also, dUTPase administered to mice, produced sickness behaviors known to be induced by some of the cytokines we showed to be upregulated. A subsequent paper showed that EBV-encoded dUTPase can enhance production of proinflammatory cytokines by monocytes/macrophages in contact with endothelial cells of blood vessels [30]. In addition, Ariza, et al demonstrated that the purified EBV-encoded dUTPase activated NFkappaB in a dose-dependent through Toll Like Receptor 2 (TLR2). Treatment of human monocyte-derived macrophages with an anti-EBV-encoded dUTPase or with an anti-TLR2 blocked the production of IL-6 [31]. Iwakiri, et al reported that EBV-encoded small RNA (EBER), which is released from EBV-infected cells, was responsible for immune activation by EBV, including release of proinflammatory cytokines [32]. A recent study (M Vera, MA Fletcher, C Cuba, L Garcia, N Klimas, presented to the International Association for Chronic Fatigue Syndrome/Myalgic Encephalitis, Reno, NV, March, 2009) reported that the anti-viral and immuno-modulatory drug, inosine pranobex, led to significant improvement in the clinical scores of 61 patients treated for 6 months. Immune activation was decreased, NK cell activity was improved and titers of anti-Epstein Barr Virus Viral Capsid Antigen IgG were significant decreased. Antibody titers to Human Herpes Virus 6 were unchanged. A larger randomized trial would seem appropriate.

According to ROC analysis, plasma IL-5 was best at distinguishing CFS cases from controls, with the highest percentage difference from the median of normal and the largest AUC. We recently reported elevation of IL-5 in the supernatants of mitogen-stimulated cultured lymphocytes from Gulf War Illness (GWI) cases compared to controls [33]. The symptoms of GWI are similar to those reported in CFS. Three other cytokines with AUC values consistent with good potential as biomarkers were LTα, IL-4 and IL-12. Less promising as systemic markers of CFS, but with AUC significantly different in cases compared to controls, were IL-6, IL-15, IL-13, IL-1α and IL-1β.

The cytokine changes observed between CFS patients and healthy, matched controls are likely to be indicative of immune activation and inflammation. Fibromyalgia, GWI, rheumatologic disorders and multiple sclerosis may have similar cytokine patterns. Future research will be required to determine if the cytokine patterns associated with CFS cases are similar or distinct from other complex, chronic and poorly understood illnesses.

Obvious limitations of this study are that the samples represent a single point in time and a single gender. The parent protocol, from which the CFS samples were gathered, is a larger longitudinal study. Subjects are followed over 18 months and sample collection includes times of relative symptom remission or exacerbation. Completion of the study will allow the correlation of CFS related symptoms and other immune markers with the cytokine patterns. CFS is a condition that affects women in disproportionate numbers. The larger study will have sufficient power to allow the study of cytokine patterns in men with CFS. As Broderick and colleagues have pointed out, markers of immune status tend to be highly variable and context-specific leading to inconsistent biomarker lists [34]. These indicators are parts of a complex and integrated system and their inter-dependency must be addressed. Accordingly, we are currently engaged in combining the proteomic and genomic data on cytokines with other immunologic and neuroendocrine markers, both proteomic and genomic, in order to map the network structure of neuroendocrine-immune interaction in CFS. We will focus on identifying associations between nodes that are differentially expressed across disease group and controls.

The finding of cytokine imbalances in the peripheral blood compartment has implications for physiological and psychological function changes. The decreased natural killer (NK) cell cytotoxic and lymphoproliferative activities and increased allergic and autoimmune manifestations in CFS would be compatible with the hypothesis that the immune system of affected individuals is biased towards a T- helper (T_H_) 2 type, or humoral immunity-oriented cytokine pattern. The elevations in LTα, IL-1α, IL1β and IL-6 indicate inflammation, likely to be accompanied by autoantibody production, inappropriate fatigue, myalgia and arthralgia, as well as changes in mood and sleep patterns.

## Conclusion

This is study is among the first in the CFS literature to report the plasma profiles of a reasonably large panel of cytokines assessed simultaneously by multiplex technique. Cytokine abnormalities appear to be common in CFS. Several showed promise as potential biomarkers. The changes from the normal condition indicate immune activation and inflammation - and point to potential therapeutic strategies. The results imply a disorganized regulatory pattern of T_H_1 function, critical to antiviral defense. The data from this study support a T_H_2 shift, pro-inflammatory cytokine up regulation and down regulation of important mediators of cytotoxic cell function.

## Competing interests

The authors declare that they have no competing interests.

## Authors' contributions

MAF and NGK conceived of the study, participated in its design, coordination, performed the statistical analysis and drafted the manuscript; NGK and SL participated in patients' diagnosis and assessment; ZB participated in subject recruitment and data management; XRZ carried out the immunoassays. All authors read and approved the final manuscript.

## Supplementary Material

Additional file 1 Coordinates of the curves for those cytokines with AUC that indicated good biomarker material. Click here for file
