# Supplementary material for: Plasma cytokines in women with chronic fatigue syndrome
Source: J Transl Med. 2009 Nov 12;7:96. doi: 10.1186/1479-5876-7-96 (PMC2779802; doi:10.1186/1479-5876-7-96)
Supplement: Additional file 1 — Coordinates of the curves for those cytokines with AUC that indicated good biomarker material. [file 1479-5876-7-96-S1.doc]

# Additional file

### Additional file 1 – Coordinates of the curves for those cytokines with AUC that indicated good biomarker potential.

| **Coordinates of the Curve** | | | |
| --- | --- | --- | --- |
| Test Result Variable(s) | Positive if Greater Than or Equal To^a^ | Sensitivity | 1 - Specificity |
| plasmaIL12 | -.3820 | 1.000 | 1.000 |
|  | .8080 | .975 | 1.000 |
|  | 1.1600 | .950 | 1.000 |
|  | 1.3620 | .950 | .983 |
|  | 1.4032 | .950 | .966 |
|  | 1.4060 | .950 | .949 |
|  | 1.4358 | .950 | .932 |
|  | 1.4725 | .950 | .915 |
|  | 1.4865 | .925 | .915 |
|  | 1.4970 | .925 | .898 |
|  | 1.5242 | .925 | .881 |
|  | 1.5552 | .925 | .864 |
|  | 1.6085 | .925 | .847 |
|  | 1.6590 | .900 | .847 |
|  | 1.6655 | .875 | .831 |
|  | 1.6690 | .875 | .814 |
|  | 1.6955 | .875 | .797 |
|  | 1.7240 | .875 | .780 |
|  | 1.7300 | .875 | .763 |
|  | 1.7465 | .875 | .746 |
|  | 1.7655 | .875 | .729 |
|  | 1.7822 | .875 | .712 |
|  | 1.8043 | .875 | .695 |
|  | 1.8202 | .875 | .678 |
|  | 1.8468 | .875 | .661 |
|  | 1.8680 | .875 | .644 |
|  | 1.8732 | .875 | .627 |
|  | 1.8852 | .875 | .610 |
|  | 1.9070 | .875 | .593 |
|  | 1.9442 | .875 | .576 |
|  | 1.9732 | .875 | .559 |
|  | 1.9912 | .875 | .542 |
|  | 2.0055 | .850 | .542 |
|  | 2.0157 | .825 | .542 |
|  | 2.0292 | .825 | .525 |
|  | 2.0338 | .825 | .508 |
|  | 2.0590 | .825 | .475 |
|  | 2.0945 | .825 | .458 |
|  | 2.1090 | .825 | .441 |
|  | 2.1162 | .825 | .424 |
|  | 2.1250 | .825 | .407 |
|  | 2.1872 | .825 | .390 |
|  | 2.2645 | .800 | .390 |
|  | 2.2905 | .800 | .373 |
|  | 2.3020 | .800 | .356 |
|  | 2.3085 | .775 | .356 |
|  | 2.3290 | .775 | .339 |
|  | 2.3650 | .750 | .339 |
|  | 2.4037 | .750 | .322 |
|  | 2.4387 | .750 | .305 |
|  | 2.4520 | .750 | .288 |
|  | 2.4615 | .750 | .271 |
|  | 2.4830 | .750 | .254 |
|  | 2.5218 | .750 | .237 |
|  | 2.5482 | .750 | .220 |
|  | 2.5592 | .725 | .220 |
|  | 2.5792 | .700 | .220 |
|  | 2.6050 | .700 | .203 |
|  | 2.6725 | .700 | .186 |
|  | 2.7635 | .675 | .186 |
|  | 2.9078 | .675 | .169 |
|  | 3.1967 | .650 | .169 |
|  | 3.5290 | .650 | .153 |
|  | 3.7218 | .625 | .153 |
|  | 3.7760 | .625 | .136 |
|  | 3.8460 | .600 | .136 |
|  | 3.9460 | .575 | .136 |
|  | 4.0402 | .550 | .136 |
|  | 4.1258 | .525 | .136 |
|  | 4.3272 | .500 | .136 |
|  | 4.5862 | .500 | .119 |
|  | 4.8182 | .475 | .119 |
|  | 5.0038 | .475 | .102 |
|  | 5.1560 | .450 | .102 |
|  | 5.2958 | .425 | .102 |
|  | 5.3450 | .425 | .085 |
|  | 5.4228 | .400 | .085 |
|  | 5.4820 | .375 | .085 |
|  | 5.4883 | .350 | .085 |
|  | 5.6672 | .325 | .085 |
|  | 5.9655 | .300 | .085 |
|  | 6.3735 | .275 | .085 |
|  | 7.1028 | .250 | .085 |
|  | 7.7225 | .225 | .085 |
|  | 8.2660 | .225 | .068 |
|  | 8.8215 | .200 | .068 |
|  | 9.6382 | .175 | .068 |
|  | 10.6797 | .150 | .068 |
|  | 12.0590 | .150 | .051 |
|  | 14.0240 | .125 | .051 |
|  | 15.4095 | .125 | .034 |
|  | 16.3205 | .125 | .017 |
|  | 18.0973 | .100 | .017 |
|  | 19.3705 | .075 | .017 |
|  | 25.7792 | .075 | .000 |
|  | 35.3595 | .050 | .000 |
|  | 75.5120 | .025 | .000 |
|  | 113.4740 | .000 | .000 |
| plasmaIL4 | -1.0000 | 1.000 | 1.000 |
|  | .0185 | .975 | 1.000 |
|  | .0610 | .975 | .983 |
|  | .1035 | .975 | .966 |
|  | .1335 | .975 | .949 |
|  | .1648 | .975 | .932 |
|  | .1967 | .975 | .915 |
|  | .2198 | .975 | .898 |
|  | .2350 | .975 | .881 |
|  | .2510 | .975 | .864 |
|  | .2748 | .975 | .847 |
|  | .2955 | .975 | .831 |
|  | .3060 | .975 | .814 |
|  | .3180 | .975 | .797 |
|  | .3345 | .975 | .780 |
|  | .3450 | .975 | .763 |
|  | .3500 | .975 | .746 |
|  | .3525 | .975 | .729 |
|  | .3565 | .975 | .712 |
|  | .3715 | .975 | .695 |
|  | .3855 | .975 | .678 |
|  | .3885 | .950 | .678 |
|  | .3960 | .950 | .661 |
|  | .4045 | .950 | .644 |
|  | .4095 | .950 | .627 |
|  | .4200 | .950 | .610 |
|  | .4292 | .950 | .593 |
|  | .4310 | .950 | .576 |
|  | .4488 | .950 | .559 |
|  | .4712 | .950 | .542 |
|  | .4842 | .950 | .525 |
|  | .4960 | .925 | .525 |
|  | .5005 | .925 | .508 |
|  | .5057 | .900 | .508 |
|  | .5117 | .900 | .492 |
|  | .5350 | .875 | .492 |
|  | .5635 | .875 | .475 |
|  | .5705 | .875 | .458 |
|  | .5728 | .875 | .441 |
|  | .5758 | .875 | .424 |
|  | .5820 | .850 | .407 |
|  | .5900 | .850 | .390 |
|  | .6035 | .825 | .390 |
|  | .6215 | .825 | .373 |
|  | .6325 | .800 | .373 |
|  | .6385 | .800 | .356 |
|  | .6500 | .800 | .339 |
|  | .7075 | .775 | .339 |
|  | .7677 | .775 | .322 |
|  | .8208 | .775 | .305 |
|  | .8685 | .750 | .305 |
|  | .8835 | .750 | .288 |
|  | .8950 | .725 | .288 |
|  | .9130 | .700 | .288 |
|  | .9590 | .675 | .288 |
|  | .9985 | .650 | .288 |
|  | 1.0140 | .625 | .288 |
|  | 1.0250 | .625 | .271 |
|  | 1.0770 | .625 | .254 |
|  | 1.1352 | .600 | .254 |
|  | 1.1765 | .600 | .237 |
|  | 1.2157 | .600 | .220 |
|  | 1.2325 | .575 | .220 |
|  | 1.2425 | .575 | .203 |
|  | 1.2465 | .575 | .186 |
|  | 1.2545 | .575 | .169 |
|  | 1.2963 | .550 | .169 |
|  | 1.3692 | .550 | .153 |
|  | 1.5235 | .525 | .153 |
|  | 1.6510 | .500 | .153 |
|  | 1.6840 | .475 | .153 |
|  | 1.7615 | .450 | .153 |
|  | 1.8825 | .425 | .153 |
|  | 2.0025 | .400 | .153 |
|  | 2.1455 | .375 | .153 |
|  | 2.2412 | .350 | .153 |
|  | 2.2508 | .350 | .136 |
|  | 2.3430 | .350 | .119 |
|  | 2.4440 | .325 | .119 |
|  | 2.4945 | .300 | .119 |
|  | 2.8722 | .275 | .119 |
|  | 3.2352 | .275 | .102 |
|  | 3.6605 | .275 | .085 |
|  | 4.2205 | .250 | .085 |
|  | 4.4640 | .225 | .085 |
|  | 4.6192 | .200 | .085 |
|  | 4.7422 | .200 | .068 |
|  | 5.1315 | .175 | .068 |
|  | 5.5772 | .150 | .068 |
|  | 5.9328 | .150 | .051 |
|  | 6.9175 | .125 | .051 |
|  | 9.9920 | .125 | .034 |
|  | 12.8820 | .100 | .034 |
|  | 16.1370 | .075 | .034 |
|  | 21.9870 | .075 | .017 |
|  | 42.6265 | .075 | .000 |
|  | 101.6880 | .050 | .000 |
|  | 166.3840 | .025 | .000 |
|  | 190.5090 | .000 | .000 |
| plasmaLTa | -.7340 | 1.000 | 1.000 |
|  | .2888 | 1.000 | .983 |
|  | .3478 | 1.000 | .966 |
|  | .4755 | 1.000 | .949 |
|  | .5985 | 1.000 | .932 |
|  | .6370 | 1.000 | .915 |
|  | .6462 | 1.000 | .898 |
|  | .6672 | .975 | .898 |
|  | .7652 | .975 | .881 |
|  | .8822 | .975 | .847 |
|  | .9350 | .975 | .831 |
|  | .9575 | .975 | .814 |
|  | .9745 | .975 | .797 |
|  | .9980 | .975 | .780 |
|  | 1.0155 | .975 | .763 |
|  | 1.0275 | .950 | .763 |
|  | 1.0475 | .950 | .729 |
|  | 1.1390 | .950 | .712 |
|  | 1.2245 | .950 | .695 |
|  | 1.2438 | .925 | .695 |
|  | 1.3088 | .925 | .678 |
|  | 1.3898 | .925 | .661 |
|  | 1.4337 | .900 | .661 |
|  | 1.4645 | .900 | .644 |
|  | 1.4840 | .900 | .627 |
|  | 1.5350 | .900 | .610 |
|  | 1.6420 | .900 | .593 |
|  | 1.7872 | .900 | .576 |
|  | 1.8960 | .900 | .559 |
|  | 1.9240 | .900 | .542 |
|  | 1.9955 | .900 | .525 |
|  | 2.0925 | .900 | .508 |
|  | 2.1302 | .900 | .492 |
|  | 2.1882 | .900 | .475 |
|  | 2.2888 | .900 | .458 |
|  | 2.3680 | .900 | .441 |
|  | 2.4140 | .875 | .441 |
|  | 2.4920 | .875 | .424 |
|  | 2.5675 | .875 | .407 |
|  | 2.6705 | .875 | .390 |
|  | 2.7775 | .875 | .373 |
|  | 2.8550 | .875 | .356 |
|  | 2.9345 | .875 | .339 |
|  | 2.9838 | .850 | .339 |
|  | 3.0848 | .825 | .339 |
|  | 3.4928 | .825 | .322 |
|  | 3.8310 | .825 | .305 |
|  | 3.8618 | .825 | .288 |
|  | 3.9695 | .800 | .288 |
|  | 4.1152 | .800 | .271 |
|  | 4.3145 | .775 | .271 |
|  | 4.4792 | .775 | .254 |
|  | 4.5550 | .750 | .254 |
|  | 4.7498 | .725 | .254 |
|  | 4.9092 | .725 | .237 |
|  | 4.9615 | .700 | .237 |
|  | 5.1282 | .700 | .220 |
|  | 5.2585 | .675 | .220 |
|  | 5.4495 | .650 | .220 |
|  | 5.6508 | .625 | .220 |
|  | 5.9272 | .600 | .220 |
|  | 6.1935 | .575 | .220 |
|  | 6.2543 | .575 | .203 |
|  | 6.3680 | .550 | .203 |
|  | 6.7275 | .525 | .203 |
|  | 7.0455 | .525 | .186 |
|  | 7.0708 | .525 | .169 |
|  | 7.4792 | .500 | .169 |
|  | 7.8932 | .475 | .169 |
|  | 7.9232 | .450 | .169 |
|  | 8.0230 | .425 | .169 |
|  | 8.2740 | .425 | .153 |
|  | 8.5662 | .400 | .153 |
|  | 8.6990 | .375 | .153 |
|  | 8.8500 | .350 | .153 |
|  | 9.1938 | .325 | .153 |
|  | 9.3985 | .300 | .153 |
|  | 9.4748 | .300 | .136 |
|  | 10.2125 | .275 | .136 |
|  | 11.5922 | .250 | .136 |
|  | 12.5618 | .250 | .119 |
|  | 12.8548 | .250 | .102 |
|  | 13.2265 | .225 | .102 |
|  | 13.5882 | .200 | .102 |
|  | 14.9680 | .175 | .102 |
|  | 17.3862 | .150 | .102 |
|  | 19.3338 | .150 | .085 |
|  | 21.1317 | .150 | .068 |
|  | 26.2985 | .150 | .051 |
|  | 32.2240 | .150 | .034 |
|  | 34.7622 | .125 | .034 |
|  | 53.2818 | .100 | .034 |
|  | 95.1068 | .075 | .034 |
|  | 127.7562 | .050 | .034 |
|  | 143.2225 | .025 | .034 |
|  | 150.6400 | .025 | .017 |
|  | 165.8765 | .000 | .017 |
|  | 181.6780 | .000 | .000 |
| plasmaIL5 | 1.2770 | 1.000 | 1.000 |
|  | 2.3230 | 1.000 | .983 |
|  | 2.5080 | 1.000 | .966 |
|  | 2.6550 | 1.000 | .949 |
|  | 2.6875 | 1.000 | .932 |
|  | 2.7778 | 1.000 | .915 |
|  | 2.8642 | 1.000 | .898 |
|  | 2.9275 | 1.000 | .881 |
|  | 2.9865 | 1.000 | .864 |
|  | 3.0065 | 1.000 | .847 |
|  | 3.0225 | 1.000 | .831 |
|  | 3.0575 | 1.000 | .814 |
|  | 3.0802 | 1.000 | .797 |
|  | 3.1250 | 1.000 | .780 |
|  | 3.1733 | 1.000 | .763 |
|  | 3.2135 | 1.000 | .746 |
|  | 3.2562 | 1.000 | .729 |
|  | 3.2848 | 1.000 | .712 |
|  | 3.3118 | 1.000 | .695 |
|  | 3.3412 | 1.000 | .678 |
|  | 3.3762 | 1.000 | .661 |
|  | 3.3872 | 1.000 | .644 |
|  | 3.3935 | 1.000 | .627 |
|  | 3.4235 | 1.000 | .610 |
|  | 3.4505 | 1.000 | .593 |
|  | 3.4550 | 1.000 | .576 |
|  | 3.4580 | 1.000 | .559 |
|  | 3.5125 | 1.000 | .542 |
|  | 3.5845 | 1.000 | .525 |
|  | 3.7213 | 1.000 | .508 |
|  | 3.8435 | 1.000 | .492 |
|  | 3.8602 | 1.000 | .475 |
|  | 3.8820 | 1.000 | .458 |
|  | 3.9300 | 1.000 | .441 |
|  | 3.9898 | 1.000 | .424 |
|  | 4.0300 | 1.000 | .407 |
|  | 4.1103 | 1.000 | .390 |
|  | 4.2140 | 1.000 | .373 |
|  | 4.3765 | 1.000 | .356 |
|  | 4.5902 | 1.000 | .339 |
|  | 4.7225 | 1.000 | .322 |
|  | 4.7640 | .975 | .322 |
|  | 4.7963 | .975 | .305 |
|  | 4.9655 | .975 | .288 |
|  | 5.1092 | .975 | .271 |
|  | 5.1525 | .950 | .271 |
|  | 5.2958 | .950 | .254 |
|  | 5.4942 | .925 | .254 |
|  | 5.5970 | .900 | .254 |
|  | 5.6228 | .900 | .237 |
|  | 5.7647 | .875 | .237 |
|  | 5.9415 | .875 | .220 |
|  | 6.0192 | .850 | .220 |
|  | 6.1202 | .825 | .220 |
|  | 6.2180 | .825 | .203 |
|  | 6.2660 | .800 | .203 |
|  | 6.3148 | .775 | .203 |
|  | 6.3538 | .750 | .203 |
|  | 6.4322 | .725 | .203 |
|  | 6.5560 | .700 | .203 |
|  | 6.6420 | .700 | .186 |
|  | 6.7462 | .675 | .186 |
|  | 6.8275 | .650 | .186 |
|  | 6.8708 | .625 | .186 |
|  | 6.9528 | .600 | .186 |
|  | 7.0022 | .575 | .186 |
|  | 7.0467 | .550 | .186 |
|  | 7.1155 | .525 | .186 |
|  | 7.2092 | .525 | .169 |
|  | 7.3662 | .500 | .169 |
|  | 7.4970 | .475 | .169 |
|  | 7.6692 | .450 | .169 |
|  | 7.8625 | .450 | .153 |
|  | 7.9638 | .450 | .136 |
|  | 8.0293 | .425 | .136 |
|  | 8.0862 | .400 | .136 |
|  | 8.1618 | .375 | .136 |
|  | 8.2145 | .375 | .119 |
|  | 8.2390 | .350 | .119 |
|  | 8.2562 | .325 | .119 |
|  | 8.3608 | .300 | .119 |
|  | 9.0415 | .275 | .119 |
|  | 9.7123 | .275 | .102 |
|  | 9.9222 | .250 | .102 |
|  | 10.2718 | .225 | .102 |
|  | 10.8312 | .200 | .102 |
|  | 11.2712 | .200 | .085 |
|  | 11.5250 | .175 | .085 |
|  | 12.0545 | .150 | .085 |
|  | 12.9765 | .150 | .068 |
|  | 15.1232 | .125 | .068 |
|  | 16.7448 | .100 | .068 |
|  | 17.3555 | .075 | .068 |
|  | 23.6118 | .075 | .051 |
|  | 29.5612 | .075 | .034 |
|  | 31.1830 | .050 | .034 |
|  | 39.8700 | .050 | .017 |
|  | 51.4578 | .025 | .017 |
|  | 107.8112 | .025 | .000 |
|  | 160.9315 | .000 | .000 |
| The test result variable(s): plasmaIL12, plasmaIL4 has at least one tie between the positive actual state group and the negative actual state group. | | | |
| a. The smallest cutoff value is the minimum observed test value minus 1, and the largest cutoff value is the maximum observed test value plus 1. All the other cutoff values are the averages of two consecutive ordered observed test values. | | | |
